# Cross-Risk Between Tuberculosis and COVID-19 in East Java Province, Indonesia: An Analysis of Tuberculosis and COVID-19 Surveillance Registry Period 2020–2022

**DOI:** 10.7759/cureus.44857

**Published:** 2023-09-07

**Authors:** Satiti Palupi, Virasakdi Chongsuvivatwong, Asik Surya, Suyanto Suyanto, Ponlagrit Kumwichar

**Affiliations:** 1 Department of Epidemiology, Prince of Songkla University, Hat Yai, THA; 2 Department of Communicable Disease, East Java Provincial Health Office, Surabaya, IDN; 3 Department of Direct Communicable Disease Prevention and Control, Ministry of Health Republic of Indonesia, Jakarta, IDN; 4 Faculty of Medicine, Riau University, Pekanbaru, IDN

**Keywords:** tuberculosis (tb), covid-19, surveillance registry, cross-risk, standardized morbidity ratio

## Abstract

Introduction: Tuberculosis (TB) and COVID-19 are highly transmissible diseases and pose a serious risk to public health. Unfortunately, information on cross-risk between the two diseases was still sparse. Our main objective was to estimate the excess risk among TB patients in getting COVID-19 infection and vice versa.

Methods: The study design was a series of analyses of existing data from TB and COVID-19 registries in East Java Province, Indonesia. The study period was from January 2020 to June 2022. Case-by-case data for this study were obtained from the registration systems for TB and COVID-19 in separate databases. In comparing risk across different groups, adjusting for differences in risk factors that influence the outcome was essential. We overcame this problem by employing a standardized morbidity ratio.

Results: Among 92,424 newly diagnosed TB patients, 1,326 were subsequently infected with COVID-19 during the study period, compared with 1,679 expected. The standardized morbidity ratio (95% confidence interval) was 72.61% (60.19%, 85.03%). Among 635,946 newly diagnosed COVID-19-infected patients, 987 subsequently got active TB during the study period against 1,679 expected. The standardized morbidity ratio (95% confidence interval) was 55.33% (49.24%, 61.42%).

Conclusion: There was no evidence of excess risk in either direction, the excess risk among TB patients in getting COVID-19 infection and vice versa.

## Introduction

Tuberculosis (TB) is caused by *Mycobacterium tuberculosis* (M.tb), which spreads from human to human through airborne particles [1]. COVID-19 is an infectious disease caused by the severe acute respiratory syndrome coronavirus 2 (SARS-CoV-2). It also spreads from individual to individual by breathing droplets from coughing and sneezing via close contact [2].

TB and COVID-19 have symptoms and transmission pathways similar to each other. Furthermore, they share clinical and radiological similarities, although they are not all the same [3]. A genetic cross-reaction between TB and COVID-19 has been identified [4]. Co-infection with TB may occur in patients with COVID-19 who have not fully recovered due to the weakened host immune system [5,6]. TB and COVID-19 can lead to worse outcomes and more severe conditions if not identified and treated promptly, thus necessitating immediate attention. Both pathogens may cause an imbalance of inflammatory responses in the immune system, and a common dysregulation of the immune response is associated with an increased risk of disease severity and progression in both diseases [7]. Generally, one infection may present an additional risk of acquiring another infection due to damage to the respiratory defense mechanism [8]. However, there is no evidence of a sequential infection between TB and COVID-19. In this study, we want to analyze secondary data from two different disease register systems, namely TB and COVID-19, to find out the sequential infection between TB and COVID-19 at the province level, which has never had a study like this before.

In Indonesia, the first case of COVID-19 was reported on March 2nd, 2020. Meanwhile, COVID-19 cases in East Java were first announced on March 17th, 2020. The TB notification rate was sharply reduced during the peak of the COVID-19 pandemic. Indonesia is a high-TB burden country with an estimated incidence of 312 per 100,000 population in 2019 [9]. It was also badly affected by the COVID-19 pandemic in 2020. At that time, the registry of COVID-19 patients with confirmed laboratory tests from reverse transcription-polymerase chain reaction (RT-PCR) had already been established. So had been the registry of patients with confirmed TB. This opened the opportunity to test the hypothesis that one disease modifies the risk for the other. If this is confirmed, we would need special close observation for patients with TB or COVID-19 to avoid getting the other infection.

Information about TB and COVID-19 is still sparse. There is evidence that the occurrence of the COVID-19 pandemic significantly reduces notification of TB [10]. However, studies using a standardized morbidity ratio to determine whether TB patients have a lower or higher risk of contracting COVID-19 and vice versa are rare. In comparing risk across different groups, adjusting for differences in risk factors influencing the outcome is essential. In population databases, behavior and other risk factors are not available. Age and sex are usually the two factors to be standardized to remove their confounding effects. People in a large area are not a homogeneous population at risk. Therefore, the comparison should be first made with the same sub-area with somewhat the same population at risk. These results can then be pooled in a proper statistical fashion.

East Java Province has a population of about 40 million with an estimated population density of 831 people per km^2^. It is divided into 38 districts. Our analysis was first conducted at the district level before pooling into the provincial level. If the increase in risk were not homogeneous, we would then identify district factors influencing the risk, which can improve our understanding of the contextual effects of the district. Our main objective was to estimate the excess risk among TB patients in getting COVID-19 infection. On the other hand, we also aimed to estimate the excess risk among COVID-19 patients in getting active TB.

## Materials and methods

Study design

The study design was a series of analyses of existing data on the TB and COVID-19 registry in East Java, Indonesia. The dataset analyzed covers the period from January 2020 to June 2022.

Nature of the dataset

Permission for analysis of the case-by-case data for this study was granted by the registration systems, namely, Sistem Informasi Tuberculosis (SITB) for TB and New All Record (NAR) for COVID-19 developed by the Ministry of Health, Republic of Indonesia. They were in separate databases. Here, we then had two cohorts. The first cohort was new TB diagnosed for all cases and registered in the TB registry. The outcome variable was whether they eventually got the COVID-19 infection, which was detected in the COVID-19 registry (henceforth, in this article, it will be written as TB followed by COVID-19). The second cohort was those who started with having COVID-19 confirmed by RT-PCR. The outcome variable is whether they eventually got active TB, which we can detect from the TB registry (henceforth, in this article, it will be written as COVID-19 followed by TB).

The duration of TB followed by COVID-19 or COVID-19 followed by TB in this study was set at least 25 days after the first disease. This arbitrary 25 days was assumed to be the shortest possible period to get exposure to the second infection after having the first one [11,12]. Omitting the 24 days would allow us to avoid Berkson’s bias or bias in detecting other diseases (such as TB) when the patient is admitted for treatment of the index disease (COVID-19).

The inclusion criteria for the records in both datasets are Indonesian citizens with valid identity documents (ID). Residing in East Java Province. The dataset was further checked to exclude duplicated citizen IDs in the same database. If a person moved from one district to another after getting the first infection, they would be included as citizens of the first district. The population statistics of each of the 38 districts of East Java were obtained from the National Statistic Office.

The measure of excess risk

In this study, we chose the standardized morbidity ratio as the measure of excess risk. For epidemiological studies, interest often lies in comparing the morbidity rate of a certain cohort with that of the general population. In such circumstances, a rough comparison of morbidity rates may be distorted by differences in age and sex [13]. We overcame this problem by employing the standardized morbidity ratio, which is described in detail below.

The standardized morbidity ratio was usually calculated using stratification by age group and sex-specific categories [14]. The formula of the standardized morbidity ratio was:

Standardized morbidity ratio = ∑(*d_j_*)/∑ (*n_j_ λ_j_*) = D/E

Standardized morbidity ratio compared the observed number of diseases in the cohort with an expected number obtained by applying the standard rates to the cohort age, sex, and district stratum. _j_ was the age-sex-district stratum, *d_j_* was the observed disease in the cohort in stratum *_j_*, *n_j_* was the cohort population in each stratum, *λ_j_* was the incidence (rate) among the general population in each stratum, D was denoted as the total observed number of disease cases in the study cohort from a specified cause (total observed disease in cohort stratum *_j_* (*d_j_*)), and E was the expected number calculated from the population [15].

The standardized morbidity ratio was expressed as a percentage after multiplying by 100. The standardized morbidity ratio interpretation was as follows:

a. Standardized morbidity ratio = 100% indicated the group had the same rate of disease as that in the population.

b. Standardized morbidity ratio > 100% denoted higher risk in the group than in the population.

c. Standardized morbidity ratio < 100% indicated the opposite [14].

The main stratification variables were age (in five-year intervals), sex, and districts. Since East Java Province has a large population of around 40 million, we examined the standardized morbidity ratio of 38 districts. For a relatively short study period of one to two years, the pandemic was very dynamic. The members of these 40 million populations changed their status over time. It would be unnecessarily complicated to compute the denominator over these 38 districts for each age group and sex for each month. So, we followed conventional standardized morbidity ratio analysis by not including time as a stratification factor.

Data analysis 

We analyzed the standardized morbidity ratio with R version 4.0.2 (R Foundation for Statistical Computing, Vienna, Austria) and modified it from a spreadsheet template published by Neyeloff et al [16]. This allowed us to obtain the estimate of the standardized morbidity ratio, the 95% confidence interval, the level of heterogeneity (I^2^), the random effect level of heterogeneity (I^2^), and the forest plot. If the meta-analysis showed heterogeneity among districts, we further performed meta-regression to test the effects on the standardized morbidity ratio of population size, population density, and the region where the district was located.

## Results

Population size in each district

**Figure 1:**
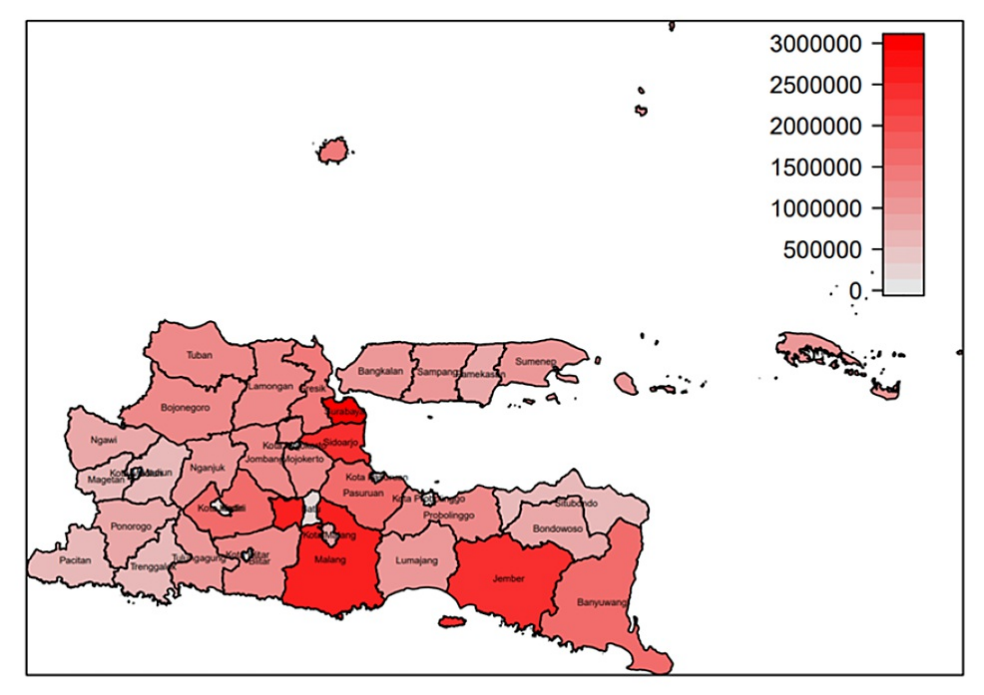
Population size in 38 districts in East Java Province.

Figure 1 shows the map of East Java with population size. The four districts with more than two million population (as marked by a dark red color) were Surabaya city, Malang, Jember, and Sidoarjo. The largest population was in Surabaya city, and the smallest population was in Mojokerto city.

Correlation between TB and population density, COVID-19 and population density, and TB and COVID-19

**Figure 2:**
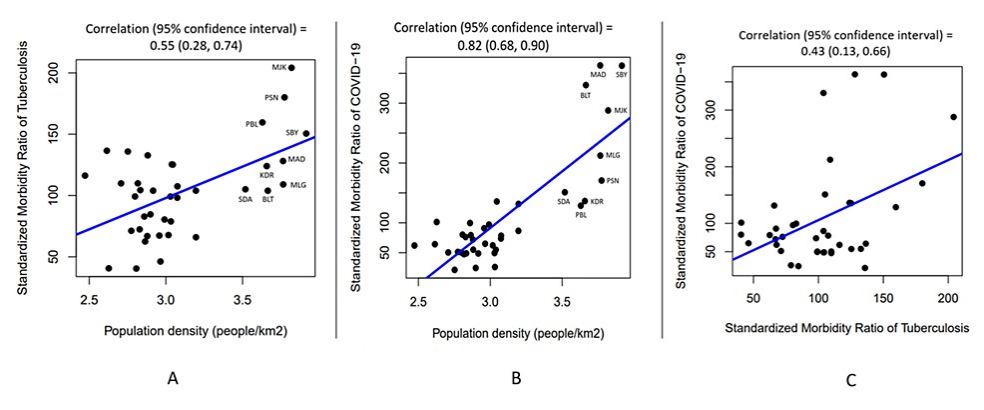
Correlation between population density and standardized morbidity ratio of tuberculosis (A), correlation between population density and standardized morbidity ratio of COVID-19 (B), and correlation between standardized morbidity ratio between the two diseases among 38 districts (C).

Figures 2A, 2B show there is a significant positive correlation between each disease and population density: correlation (95% confidence interval) = 0.55 (0.28, 0.74) and 0.82 (0.68, 0.90), respectively. Nine districts with high population density had a high standardized morbidity ratio for both TB and COVID-19. They were Surabaya city (SBY), Mojokerto city (MJK), Pasuruan city (PSN), Malang city (MLG), Madiun city (MAD), Blitar city (BLT), Kediri city (KDR), Probolinggo city (PBL), and Sidoarjo (SDA).

Figure 2C shows the correlation between TB disease and COVID-19 infection. The two variables moved in the same direction. As TB disease increased, COVID-19 also tends to increased. The correlation was moderately positive with correlation (95% confidence interval) = 0.43 (0.13, 0.66), P value < 0.05.

Standardized morbidity ratio of TB followed by COVID-19 and standardized morbidity ratio of COVID-19 followed by TB

There were 92,424 unique records in the TB registry and 635,946 in the COVID-19 registry. Combining the unique records of those two registries, there were 2,313 people affected by both diseases at least 25 days apart. 1,326 of them were affected by COVID-19 after TB (TB followed by COVID-19), and 987 were in reverse order (COVID-19 followed by TB).

**Figure 3:**
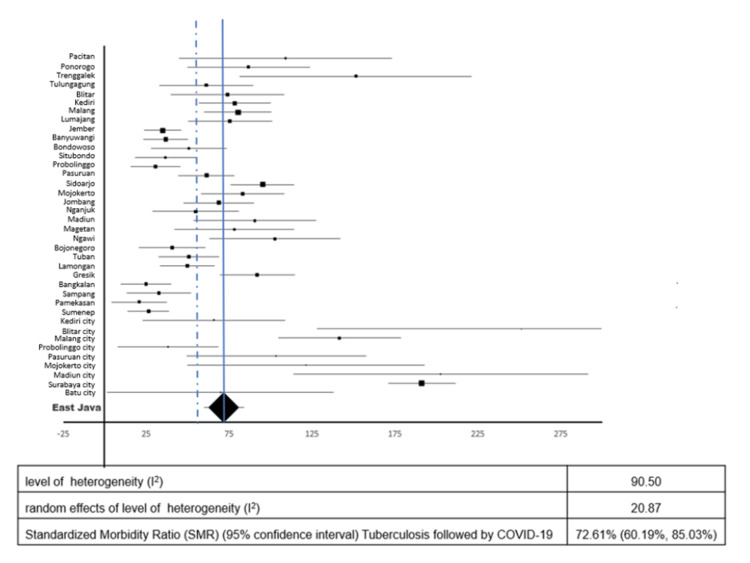
Forest plot standardized morbidity ratio of tuberculosis followed by COVID-19.

**Figure 4:**
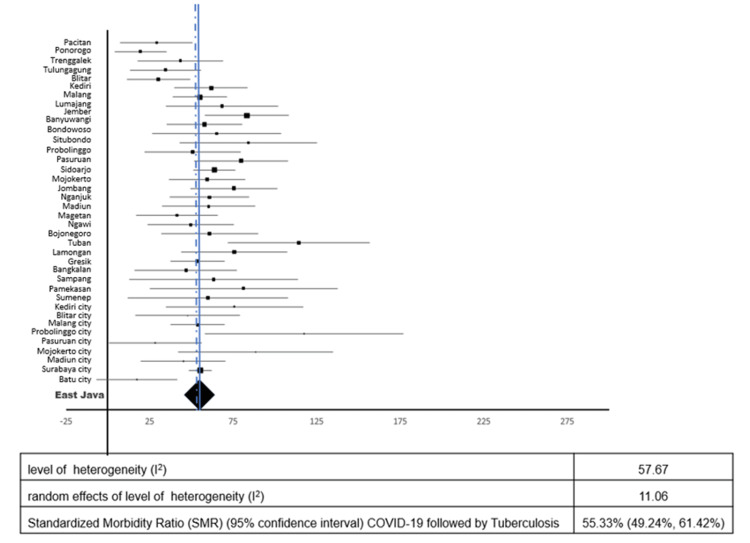
Forest plot standardized morbidity ratio of COVID-19 followed by tuberculosis.

Figures 3, 4 show TB followed by COVID-19 and COVID-19 followed by TB, respectively. The respective pooled standardized morbidity ratios (95% confidence interval) were 72.61% (60.19%, 85.03%) and 55.33% (49.24%, 61.42%), and the respective levels of heterogeneity (I^2^) were 90.5% and 57.7%. After fitting random effects models, the respective levels of heterogeneity (I^2^) were reduced to 20.9% and 11.1%, respectively. The median time for TB followed by COVID-19 during the study period was 241 days (IQR, 99-421.75 days), and that for COVID-19 followed by TB was 179 days (IQR, 79-310 days).

Meta-regression standardized morbidity ratio of TB followed by COVID-19 and standardized morbidity ratio of COVID-19 followed by TB

Tables 1, 2 show meta-regression results that predict the standardized morbidity ratio of TB followed by COVID-19 and the standardized morbidity ratio of COVID-19 followed by TB from population size, population density, and region. For TB, followed by COVID-19, all predictors were statistically significant. The LR-test P value for the region < 0.001 (not shown in the table). Regions Jember and Pamekasan have a significantly lower standardized morbidity ratio than the referent Region Bojonegoro, which was omitted as the referent level. For COVID-19 followed by TB, neither of the population predictors was statistically significant, but for the LR-test, the P value for the region was <0.001. Region Madiun had a significantly lower standardized morbidity ratio than the referent Region Bojonegoro.

**Table 1:** Meta-analysis regression of the standardized morbidity ratio of TB followed by COVID-19. TB: tuberculosis.

Predictors	Relative risk	Exp (standard error)	t-value	P-value
Population size	1.36	1.13	2.54	0.02
Population density	1.18	1.03	5.22	0.00
Regions				
Bojonegoro	Referent			
Jember	0.66	1.18	−2.48	0.02
Madiun	1.49	1.26	1.69	0.10
Malang	1.01	1.26	0.06	0.95
Pamekasan	0.60	1.18	−2.99	0.01

**Table 2:** Meta-analysis regression of the standardized morbidity ratio of COVID-19 followed by TB. TB: tuberculosis.

Predictors		Relative risk	Exp (standard error)	t-value	P-value
Population size		1.09	1.09	1.03	0.31
Population density		0.99	1.02	−0.30	0.76
Regions					
Bojonegoro	Referent				
Jember		0.95	1.25	−0.23	0.82
Madiun		0.52	1.19	−3.67	0.00
Malang		0.66	1.22	−2.03	0.05
Pamekasan		0.78	1.23	−1.20	0.24

## Discussion

Our standardized morbidity ratio analysis shows that there was a diversity in risk of getting TB and COVID-19 over different districts of East Java. Both diseases increased in high population density areas with a moderate positive correlation. Our meta-analysis suggested that in the population, people affected by one disease were less likely to get the other disease compared to the general population. The risk of getting COVID-19 after TB significantly increased in higher population density districts, but this was not so for those getting TB after COVID-19 infection.

Compared to our findings, previous studies in Bangladesh and India reported a positive correlation between TB cases and population density. Correlation coefficients (*r*) were 0.45 and 0.76, respectively [17,18]. Meanwhile, in another study in Brazil and Malaysia, there was a positive correlation between COVID-19 and population density, with correlation coefficients (*r*) of 0.33 and 0.78, respectively [19,20].

Although there is a positive correlation between TB and population density, we can see that it is smaller if we compare the correlation between COVID-19 and population density, so it can be seen that the actual number of TB notifications is likely higher than the current number of TB notifications.

This and previous findings that the standardized morbidity ratio of these two diseases is correlated with population density could be explained by the susceptible infectious recovered (SIR) theory [21]. Infectious disease spread depends on the contact rate between susceptible and infected people. This contact rate is high in congested areas. The condition is aggravated by poor ventilation in the urban area as well as traveling activity [22-24]. Densely populated urban areas are considered places with a higher risk of transmission, including crowded and slum settlements [25]. Studies in China show that overcrowding and poor ventilation contribute to the spread of airborne infectious diseases [26]. Human mobility is mostly in high populations and plays a crucial role in the temporal and spatial spreading of infectious diseases [20].

COVID-19 is an acute disease with a short incubation period compared to TB [12]. From the previous study, we get information that a large percentage of active TB cases were reactivation cases from latent TB infection [27]. In East Java, the data on the reactivation of latent TB are not yet available. This can be a reason why the effect of population density on disease transmission is stronger for COVID-19 than for TB in the general population.

We found that people affected by one disease were less likely to get the other disease compared to the general population. Several reasons can explain to these findings. The first reason can be differential notification between the diseased and the general population. The second reason is the difference in susceptibility to the disease. The third is the reduction in exposure to the second disease after getting the first one.

If identified cases are taken into account, there is a large undetected population that is carrying the disease without symptoms due to inadequate public health monitoring and testing [28]. Differential notification can occur if one with an existing disease fears getting tested for the second. There was a report that the number of people seeking medical services has drastically reduced. About 41% of patients delayed or avoided seeking medical attention due to fear of COVID-19 [29]. Self-stigma can lead to withdrawal and disengagement from health services for infectious diseases. Both fear and stigma can potentially reduce the tendency to seek medical services and counseling [30].

As mentioned in the introduction, we expected that people infected by the first disease would be more susceptible to the other due to potential mechanisms such as lymphopenia, including the depletion of TH4 cells, T-cell exhaustion (defined as progressive loss of effector function due to prolonged antigen stimulation) and cytokine storm, eventually leading to immune dysregulation [31]. Moreover, severe COVID-19 patients were often treated with high-dose corticosteroids, suppressing immune responses and possibly leading to the activation of latent TB [32]. However, in our results, a decrease in risk was found. Therefore, the difference in susceptibility would not be a plausible explanation.

The limitations of this study are not possible to cover risk behaviors in the dataset and limitations in under-reporting data, which may be different between those infected with one of these two diseases and the general population. Calculating the risk for any of these diseases in the population (the dominator part of the standardized morbidity ratio) does not need personal identification. Still, those infected with the first disease need accurate personal identification for both the first and second diseases. Inaccurate personal identification on any of the two databases will reduce the number of cases, which is the numerator of the standardized morbidity ratio, resulting in an underestimation of the excess risk. Our report of a lower risk of getting a disease after infection of the other needs to be interpreted with serious caution.

## Conclusions

Our objective was to estimate the excess risk among TB patients in getting COVID-19 infection and vice versa. In conclusion, there was no evidence of excess risk in both directions, the excess risk among TB patients in getting COVID-19 infection and vice versa.
